# Bi-rooted primary maxillary canines: a case report

**DOI:** 10.1186/s13256-019-2174-9

**Published:** 2019-08-07

**Authors:** Ali Assiry

**Affiliations:** 1 Paedatric Dentistry, Department of Preventive Dental Science, Faculty of Dentistry, Najran University, Najran, Kingdom of Saudi Arabia; 2 Saudi Board of Pediatric Dentistry, King Fahad Hospital, Medina, Kingdom of Saudi Arabia

**Keywords:** Case report, Dental anomaly, Bi-rooted primary canines, Saudi Arabia

## Abstract

**Background:**

Anomalies in primary teeth are comparatively fewer than in the permanent teeth. The presence of a primary canine with two roots is very rare. An unusual anomaly like this may lead to problems during extraction or exfoliation. Emphasis on the importance of anomalies is required for proper diagnosis and to facilitate a better treatment outcome.

**Case presentation:**

The present case report describes a case of a bilateral bi-rooted primary maxillary canines diagnosed during a radiographic examination in a 9-year-old Saudi boy. To the best of our knowledge, this is the first case of bi-rooted primary maxillary canine reported from the region of Saudi Arabia.

**Conclusion:**

This case report aims to increase awareness of the morphological alterations in primary canines and to emphasize the importance of diagnosis and radiographic examination using different angles. Clinicians should consider all the possible tooth variations during routine intra-oral and radiographic examinations to facilitate a better treatment outcome and to avoid unwanted complications.

## Background

The primary and the permanent teeth are subject to considerable variation in their form, size, number, and structure of the dental tissues. Abnormalities in tooth morphology in primary teeth are comparatively fewer than in the permanent teeth [1]. The presence of a single root in a primary canine has been described as a normal and the most common form of root morphology. However, few cases have been reported regarding the presence of a bi-rooted primary canine, the first being in 1941 [2]. It has been seen that the prevalence of bi-rooted primary canines is higher in the maxilla than in the mandible and they seem to occur bilaterally [3]. Although, the exact etiology of this anomaly is unknown, it has been suggested that an ingrowth of a tissue from Hertwig’s epithelial root sheath may be a possible cause [4]. This case report describes a case of bi-rooted primary maxillary canines occurring bilaterally. The present case report aims to increase awareness about the morphological alterations in primary canines and to emphasize the importance of diagnosis and radiographic examination using different angles.

## Case presentation

**Fig. 1:**
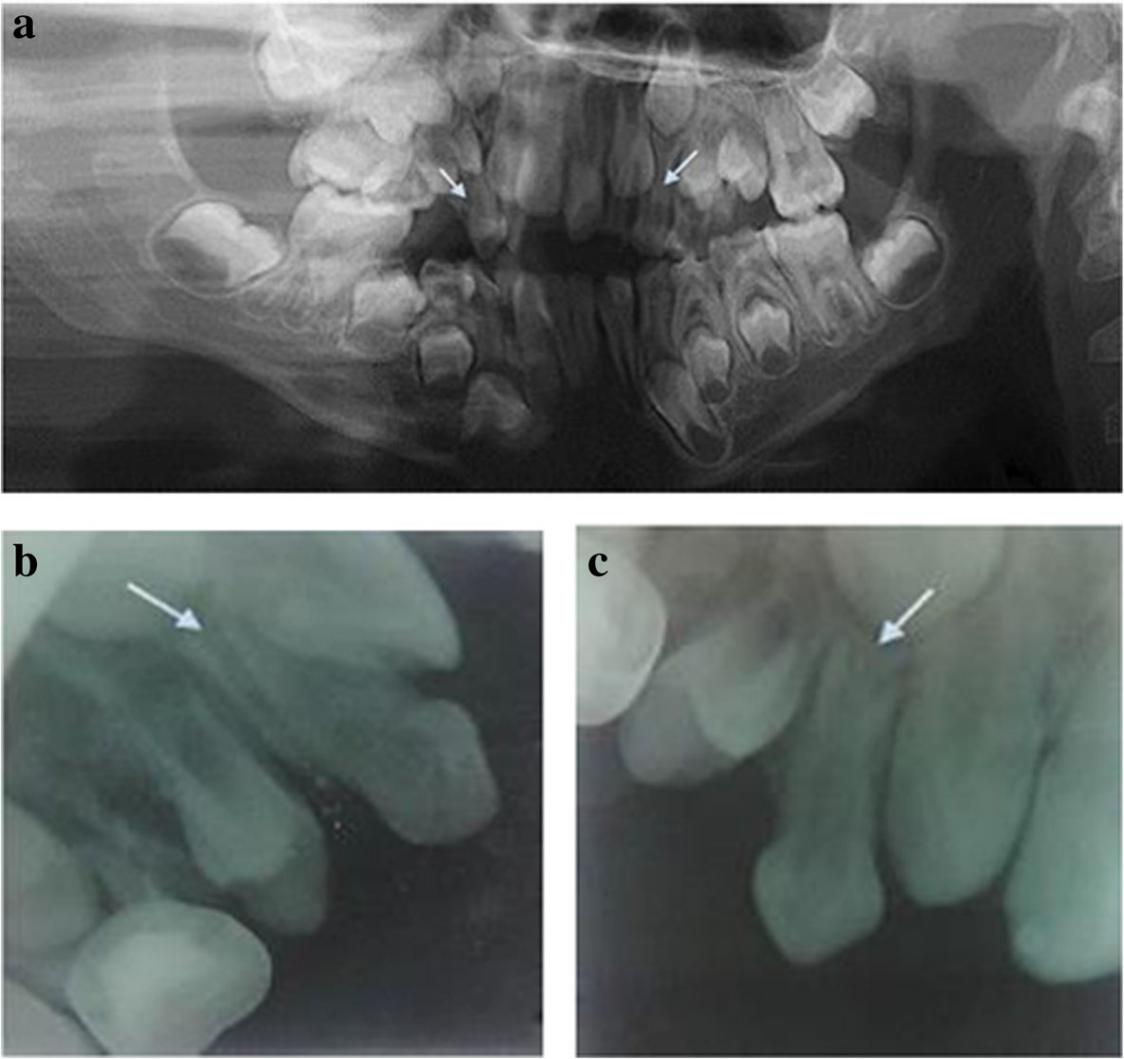
The arrows in the figure indicate (**a**) Orthopantomogram (OPG) showing Bilateral Birooted primary maxillary canines. (**b**) Periapical radiograph showing the presence of two roots in primary right maxillary canine. (**c**) Periapical radiograph showing the presence of two roots in primary left maxillary canine

**Fig. 2:**
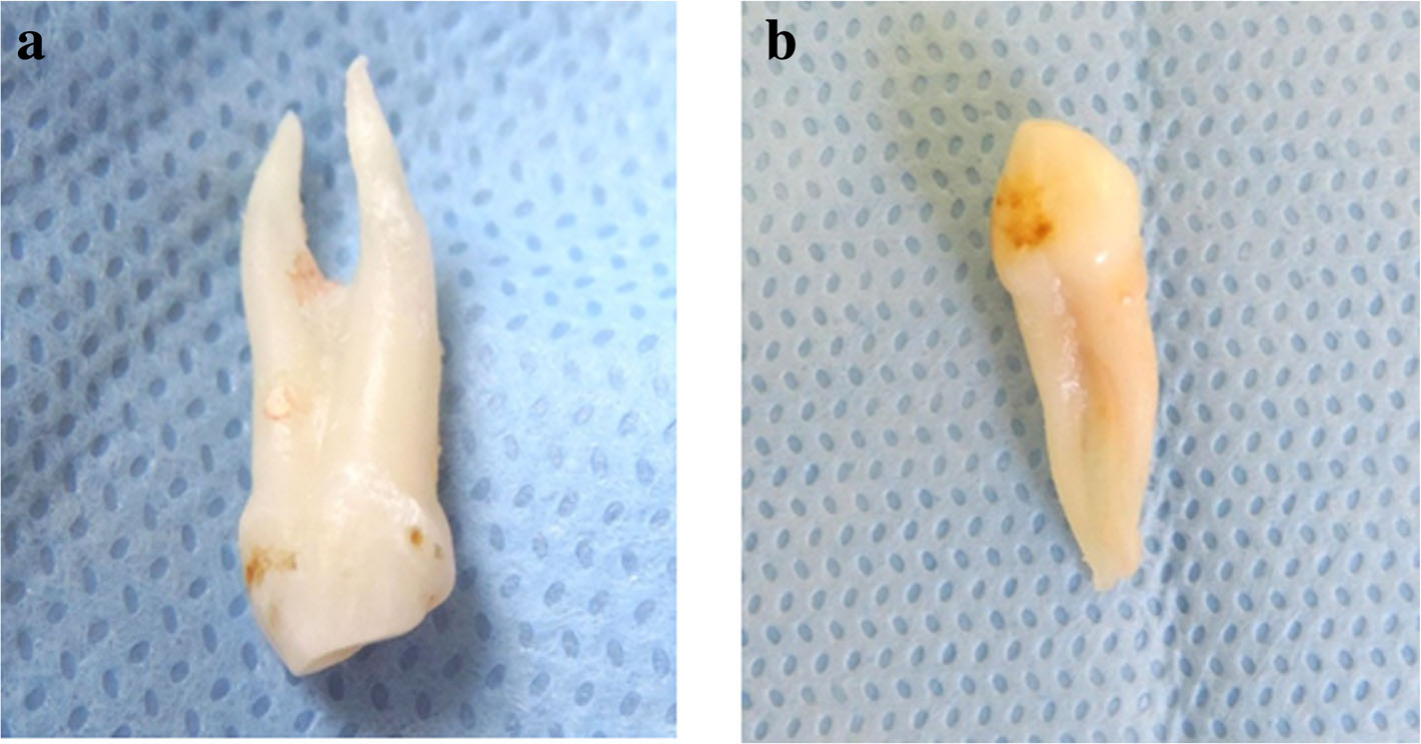
**a** Primary right maxillary canine showing two separate roots (mesial and distal). **b** Primary left maxillary canine showing two roots (mesial and distal) which are connected

On 3 April 2017, a 9-year-old Saudi boy with a complaint of missing teeth in maxillary anterior region visited the Department of Preventive Dental Science, Faculty of Dentistry, Najran University, Saudi Arabia. On 15 April 2017, he underwent a clinical examination which revealed: unerupted permanent maxillary lateral incisors; decay in tooth numbers 54, 55, 62, 64, 65, 26, 84, 74, 75, and 85 (Federation Dentaire Internationale notation); and anterior crossbite between tooth numbers 53 and 83. Radiographic examination revealed bi-rooted bilateral maxillary canines (53 and 63) (Fig. 1). On 25 April 2017, a treatment plan was made followed by: pulpotomy and stainless steel crowns in tooth numbers 55, 74, and 75; extraction of 54, 62, 64, 65, and 85; composite restoration in 84; amalgam restoration in 26; and fissure sealant was placed on 16, 36, and 46. Space evaluation and orthodontic consultation to facilitate the eruption of permanent maxillary lateral incisors (12 and 22) was done and extraction of bilateral primary canines (53 and 63) was indicated. Following extraction, the presence of two roots was confirmed by careful examination. The right primary maxillary canine had two separate roots (mesial and distal) (Fig. 2), whereas the left primary maxillary canine had two roots (mesial and distal) which were connected (Fig. 2). His parents were informed about the root anomaly and our patient was kept under careful observation to evaluate proper eruption of unerupted permanent lateral incisors (Table 1).

**Table 1:** Timeline of case

Date	Summary of visit
3 April 2017	*Clinical visit*. A 9-year-old boy visits Department of Preventive Dental Science, Faculty of Dentistry, Najran University, Saudi Arabia *Complaint*. Complaint of missing teeth in maxillary anterior region
15 April 2017	*Diagnostic visit and test*. Clinical examination revealed: • unerupted permanent maxillary lateral incisors • decay in tooth numbers 54, 55, 62, 64, 65, 26, 84, 74, 75, and 85, and • anterior crossbite between tooth numbers 53 and 83
25 April 2017	*Treatment plan*: • Pulpotomy and stainless steel crowns in tooth numbers 55, 74, and 75, • extraction of 54, 62, 64, 65, and 85, • composite restoration in 84, • amalgam restoration in 26, • and fissure sealant was placed on 16, 36, and 46. • Space evaluation and orthodontic consultation to facilitate the eruption of permanent maxillary lateral incisors (12 and 22) • Extraction of bilateral primary canines (53 and 63) was indicated
5 May 2017	*After extraction, an examination was done to find anomaly*: • The presence of two roots was confirmed by careful examination. • Right primary maxillary canine had two separate roots (mesial and distal) (Fig. 2) • Left primary maxillary canine had two roots (mesial and distal) which were connected (Fig. 2)
20 May 2017	*Follow up and conclusion*: • The patient was kept under careful observation to evaluate proper eruption of unerupted permanent lateral incisors • Clinicians should consider all the possible tooth variations during routine intra-oral and radiographic examinations to facilitate a better treatment outcome and to avoid unwanted complications

## Discussion and conclusion

A bi-rooted primary canine is an extremely rare dental anomaly with high prevalence in maxilla rather than in the mandible and it occurs more frequently in male children. To the best of our knowledge, this is the first reported case in an Arab child. A list of cases with bi-rooted primary maxillary canine from all over the world is presented in Table 2. The diagnosis and identification of tooth morphology are the main factors for an appropriate plan of treatment. A primary radiograph is significant as it helps in the identification or uncertainties of anatomical variations. Bifurcations in the roots can be seen when the X-ray has no superimposition of images [23]. In the present case, the two roots were evident in a radiograph. However, this may sometimes be difficult due to crowding of teeth. Hence, radiographic images must be cautiously analyzed to infer and recognize particulars that might propose the presence of bifurcations [24].

**Table 2:** A list of cases with bi-rooted primary maxillary canine

Author and reference	Year	Location of canine	Age	Sex	Ethnicity
Takano [5]	1941	Mandibular right	9	M	Japanese
Kurosu *et al.* [6]	1968	Maxillary right	8	F	Japanese
		Maxillary left	8	M	Japanese
		Mandibular right	8	M	Japanese
Brown [2]	1975	Bilateral maxillary	4	F	Not reported
Yasunaga *et al.*[7]	1978	Bilateral maxillary and bilateral mandibular	6	M	Japanese
Kelly [4]	1978	Bilateral maxillary	5	F	Black
Hata *et al*. [8]	1979	Bilateral mandibular	4	M	Japanese
Krolls and Donahue [9]	1980	Bilateral maxillary	5	F	Black
Chow [10]	1980	Bilateral maxillary	5	M	Black
Bryant and Bowers [11]	1982	Bilateral maxillary and bilateral mandibular	5	M	White
Bimstein and Bystrom [12]	1982	Bilateral maxillary	5	M	Black
Paulson *et al*. [13]	1985	Bilateral maxillary	9	M	Black
Jones and Hazelrigg [14]	1987	Bilateral maxillary	5	M	Black
Saravia [3]	1991	Bilateral maxillary	4	F	Black
Hayutin and Ralstrom [15]	1992	Maxillary right	4	F	Black
		Maxillary right	1	M	Black
Ott and Ball [16]	1996	Bilateral maxillary	8	M	Black
		Bilateral maxillary Bilateral mandibular	4	M	Black
			8	M	Black
Winkler and Ahmad [17]	1997	Maxillary left	4	F	Pueblo
Mochizuki *et al*. [18]	2001	Bilateral maxillary and bilateral mandibular	6	M	Japanese
Atac and Cetinguc [19]	2005	Bilateral maxillary	6	M	White
		Bilateral maxillary	6	M	White
Orhan and Sarı [20]	2006	Maxillary left	11	M	White
		Maxillary right	4	M	White
		Bilateral maxillary	6	M	White
Dhanpal and King [21]	2009	Bilateral maxillary and bilateral mandibular	15	M	Chinese
Talebi *et al*. [1]	2010	Bilateral maxillary	6	F	Iranian
Guler [22]	2012	Maxillary right	7	M	White
Present case	2017	Bilateral maxillary	9	M	Arab

The standard morphology of the primary canine includes a slender, long, and tapering root which is double the size (in length) of the crown. It has been explained that during normal root formation, at the dental organ’s cervical loop, the outer and the inner enamel epithelia multiply in the form of a double layer of cells called Hertwig’s epithelial root sheath. The inner and outer epithelia of enamel turn at the future cementoenamel junction, producing the epithelial diaphragm. The primary apical foramen is enclosed by the rim of this sheath. An unknown aspect in multi-rooted teeth stimulates continued morphodifferentiation. Tongue-like extensions of the horizontal diaphragm grow and extend toward each other, and fuse by differential growth. For every new secondary apical foramen, a root will be developed [25].

Although trauma and other disturbances may affect morphodifferentiation, improved expression of the gene starting the differential growth of Hertwig’s epithelial root sheath or a defect in the dental lamina through the initial stage of formation of the root is thought to develop double roots [20]. In the present case, the presence of two roots in primary canines bilaterally cannot be attributed to a specific cause. Hence, when an anomaly like this happens, difficulty during exfoliation or extraction may happen. The permanent canine has to reabsorb both the roots of the primary canine evenly to facilitate its normal eruption. During extraction, the clinician should confirm that the crown of the underlying permanent tooth is not trapped in order to prevent accidental removal of the underlying developing permanent tooth bud.

In conclusion, clinicians should consider all the possible tooth variations during routine intra-oral and radiographic examinations to facilitate a better treatment outcome and to avoid unwanted complications. Also, the presence of two roots in primary canines bilaterally cannot be attributed to a specific cause in the present report and further studies are needed for the proper treatment of this anomaly.

## Data Availability

Data sharing does not apply to this article as no datasets were generated or analyzed during the current study.
